# Rest Unassured: A Systematic Review on the Impacts of Daytime Napping in the Mediterranean on Health Outcomes, Quality of Life, and Lifespan

**DOI:** 10.1177/15598276261481759

**Published:** 2026-09-11

**Authors:** Liam Ellis, Bernado C. Barata, Russell Conduit, Barbora de Courten, Catherine Itsiopoulos, Gerard A. Kennedy, Ourania Kolokotroni, Rob Lawson, Maria C. Mosquera, Emmanuel Aprilakis, Achilleas Pavlou, Sophia Xenos, Yang Yap, Labros S. Sidossis

**Affiliations:** 1 Mediterranean Lifestyle Medicine Institute, Leros, Greece (LE, BCB, RC, BdC, CI, GAGAK, OK, EL, MCM, EA, AP, SX, YY, LSS); 2 School of Health and Biomedical Sciences, 5376 RMIT University, Bundoora, VIC, Australia (LE, RC, BdC, CI, GAGAK, SX, YY); 3 Departamento de Psiquiatria e Saúde Mental, 679736 Unidade Local de Saúde do Arco Ribeirinho, Barreiro, Portugal (BCB); 4 Portuguese Society of Lifestyle Medicine, Lisbon, Portugal (BCB); 5 Department of Medicine, School of Clinical Sciences, Monash University, Clayton, VIC, Australia (BdC); 6 Department of Diabetes and Vascular Medicine, Monash Health, Clayton, VIC, Australia (BdC); 7 School of Health Sciences, 121991 Cyprus University of Technology, Limassol, Cyprus (OK); 8 British Society of Lifestyle Medicine, European Lifestyle Medicine Council, World Lifestyle Medicine Organisation, East Lothian, UK (RL); 9 Department of Primary Care and Population Health, 121343 University of Nicosia Medical School, Nicosia, Cyprus (MCM); 10 Department of Global Languages and Cultures, College of Arts & Sciences, Texas A&M University, College Station, Texas, USA (EA); 11 121343 University of Nicosia Medical School, Nicosia, Cyprus (AP); 12 Department of Kinesiology and Health, School of Arts & Sciences, Rutgers University, New Brunswick, NJ, USA (LSS)

**Keywords:** daytime napping, health outcomes, quality of life, lifespan, Mediterranean lifestyle

## Abstract

Daytime napping is a culturally embedded practice in Mediterranean populations, yet its health impacts remain unclear. When assessed in an international context, the impact of napping shows mixed results. This systematic review evaluated the associations between daytime napping, health outcomes, quality of life, and lifespan in Mediterranean populations, where napping is culturally embedded. Following PRISMA guidelines, electronic searches of Embase, PsycINFO, PubMed, and SCOPUS were conducted. Eligible studies were empirical, peer-reviewed, and only included samples from the Mediterranean region. Findings were inconsistent across all health outcomes. However, there was a trend for short naps (≤30 min/day) to be associated with beneficial cardiovascular outcomes and metabolic health. In contrast, longer naps (>30 min/day) were more often associated with increased risk of cardiometabolic diseases in older adults. Interpretation was limited by methodological heterogeneity, including varied nap definitions, over-reliance on self-reports, and poor evaluation and/or adjustment for sleep disorders or excessive daytime sleepiness. These findings imply that naps should be seen as part of the broader Mediterranean lifestyle and future research should use validated, standardised assessments of napping while simultaneously accounting for sleep disorders, daytime sleepiness, and nocturnal sleep. This will lead to better insights into the role of culturally embedded napping in health.

″However, studies investigating napping outcomes were complicated by methodological heterogeneity, including varied definitions of naps, timing, duration, and frequency, as well as a lack of account of sleep disorders and daytime sleepiness.″

## Introduction

Daytime naps are short periods of sleep that occur independently from the main nocturnal sleep period.^1^ Napping has been associated with positive health outcomes,^2,3^ increased quality of life,^4^ and lifespan.^5^ The siesta, daytime napping, is a culturally embedded practice in the Mediterranean region, which encompasses over 20 countries bordering the Mediterranean Sea. Despite precise statistics on nap prevalence being difficult to ascertain, evidence suggests its continued practice across diverse age groups in the Mediterranean region.^6^ This region is consistently linked to longevity and favourable health outcomes.^7,8^ Historically, these benefits have been attributed to the Mediterranean diet (MD).^9^ However, emerging literature points to a broader constellation of health-promoting behaviours, leading to the conceptualisation of the Mediterranean lifestyle (ML), a holistic model that incorporates physical activity, social connectedness, diet, and sleep practices.^6,10-12^

Researchers have developed ML indices and questionnaires to quantify adherence.^13^ Adherence to the ML, including napping, has been associated with beneficial impacts on overall health and quality of life.^4^ Yet, existing indices often omit napping, despite its cultural significance in the Mediterranean region.^6^ This omission risks underestimating the role of napping in the health advantages attributed to the ML. Therefore, it is necessary to investigate whether napping itself exerts measurable benefits or risks for Mediterranean populations.

Evidence from outside the Mediterranean context has produced contradictory results. Some studies suggest that naps improve cognitive performance^14^ and reduce the incidence of health conditions,^3^ while others report associations with adverse outcomes such as type 2 diabetes mellitus,^15^ cardiovascular disease, and mortality.^16^ Reviews and meta-analyses similarly highlight both protective and harmful associations, which is complicated by the possibility that napping might be a result of adverse health conditions.^5,17^

Differences in participant characteristics may contribute to the mixed results reported in the literature. For example, few studies exclude individuals with chronic health conditions who are prone to excessive daytime sleepiness^18^ and take longer (>30 min/day) and more frequent naps.^2^ Dose-response meta-analyses suggest a J-curve relationship between nap duration and health outcomes, with short naps (≤30 min) linked to protective effects and longer naps (>30 min) associated with adverse outcomes such as cardiovascular risk^16^ and all-cause mortality.^5^ However, these findings were marked by substantial heterogeneity in study design and residual confounds, including participant lifestyle habits and underlying health status. Including such populations risks overstating the links between napping and adverse outcomes. For example, shift workers or older adults with chronic illness are more likely to nap to alleviate fatigue due to poor nocturnal sleep,^19^ complicating efforts to disentangle whether health outcomes are attributable to naps themselves or to the conditions that necessitate them. Clarifying whether these inconsistent associations reflect differences in nap duration and frequency, or instead arise from variation in population health status, requires focused investigation in healthy populations.

Additionally, few studies account for the cultural context of napping. In many non-Mediterranean settings, napping is often a compensatory response to fatigue, insufficient nocturnal sleep, or circadian disruption.^20,21^ These naps are not embedded within a broader lifestyle of health-promoting behaviours but occur in contexts where adverse lifestyle factors may confound associations with health. By contrast, in the Mediterranean region, naps are proactive, intentional, and socially accepted, forming part of daily routines and cultural norms.^6,12^ Neglecting to account for this cultural context risks conflating the outcomes of culturally embedded naps with those of compensatory naps practiced under less favourable conditions. Examining napping within the Mediterranean, where it is integrated into a lifestyle consistently linked with positive health outcomes, quality of life, and longevity, offers a unique assessment of its potential benefits or harms.

The primary aim of this systematic review is to evaluate the empirical evidence on the impact of daytime napping on health outcomes, quality of life, and mortality within Mediterranean populations, where napping is embedded in cultural routines and health-promoting practices. Given the mixed international literature on whether napping confers benefits or risks, this review also seeks to explore whether specific nap characteristics, such as duration and frequency, influence outcomes. The review will provide a critical evaluation of the current literature to support a culturally informed understanding of sleep as a component of the Mediterranean lifestyle and its implications for public health.

## Method

This systematic review was conducted in accordance with the Preferred Reporting Items for Systematic Reviews and Meta-Analysis (PRISMA) guidelines.^22^ The protocol for this review was registered on the PROSPERO database (CRD420251042563).

### Search Strategy

An electronic literature search on Embase, PsycINFO, PubMed, and SCOPUS was conducted on 25 June 2025, encompassing all available records up to that date without temporal filters. The following key search terms were used: (“Mediterranean”) AND (“nap” OR “siesta”) AND (“health outcomes” OR “quality of life” OR “mortality”). A comprehensive search strategy is shown in the supplementary material (Supplemental 1).

### Eligibility Criteria

Study selection criteria were established in accordance with the PICOS (Population, Intervention, Comparator, Outcome, Study type) framework. Included studies were full-text, peer-reviewed journal articles published in English.

#### Population

Eligible studies were required to focus on a healthy Mediterranean population. A healthy population was defined as individuals without diagnosed clinical conditions or health indicators suggestive of such conditions. Studies involving participants with diagnosed sleep disorders or those taking medications known to impact sleep were excluded due to the documented alterations in sleep quality compared to the general population.^23^ Studies of shift workers were excluded due to their distinct sleep patterns and higher daytime sleepiness, which could confound results.^19^ Studies of pregnant participants were excluded due to the impact of physiological and hormonal changes on sleep.^24^ Studies conducted during major disruptions (e.g. war, COVID-19 lockdowns) were excluded due to their effects on circadian rhythms and atypical sleep.^25,26^

#### Intervention

A daytime nap was defined as a short sleep during the daytime and that occurred independently from a more substantial nocturnal sleep period.^1^ Subjective (e.g. self-report) or objective (e.g. actigraphy) nap measures were considered for inclusion.

#### Comparator

Studies required a comparator group of non-nappers or nap groups differing in duration, timing, or frequency, meeting the population criteria.

#### Outcome

Eligible studies focused on the association between napping and any health outcomes, quality of life measures, or lifespan as measured by self-report, physician diagnosis, medical information database, or obituary. Studies examining the incidence of sleep disorders were excluded, as such conditions are often associated with increased daytime napping and introduce the potential for reverse causality between napping and sleep disturbance.^27^

#### Study Design

Eligible studies were those with an observational or experimental empirical study design. Non-empirical-based studies were excluded, such as literature reviews, qualitative studies, case-studies, book chapters, and conference abstracts.

### Study Selection

All records retrieved from the database searches were imported into the systematic review management platform, Covidence.^28^ Two of the authors (LE & RC) independently screened the titles and abstracts against the eligibility criteria. The full texts of included studies were sourced and independently reviewed by the 2 reviewers for inclusion. Discrepancies at both screening stages were resolved through discussion with a third reviewer (SX). Discrepancies amongst the critical appraisal assessments were resolved through consensus between the 2 assessors.

### Data Extraction

A data summary table was generated using the PICOS framework and included the following headings: author, year, country, study aim, study design, population, nap characteristics (e.g. duration, time of day), comparator group, outcome measured, and key findings. Any discrepancies were identified and resolved through discussion between assessors.

### Quality Appraisal

The methodological quality of each included study was independently assessed using the relevant Joanna Briggs Institute (JBI) Critical Appraisal Tool.^29^ The tool used corresponded to the specific study design (e.g. cohort, cross-sectional). Each tool consists of a set of criteria with a number of items designed to evaluate internal validity, bias, and methodological quality of each study. Items were rated as “yes,” “no,” “unclear,” or “not applicable.” Two reviewers (LE & RC) independently appraised each study, with discrepancies resolved by a third reviewer (SX). Overall quality scores were converted into percentages based on satisfied vs total appraisal items.

## Results

### Study Selection

**Figure 1.**
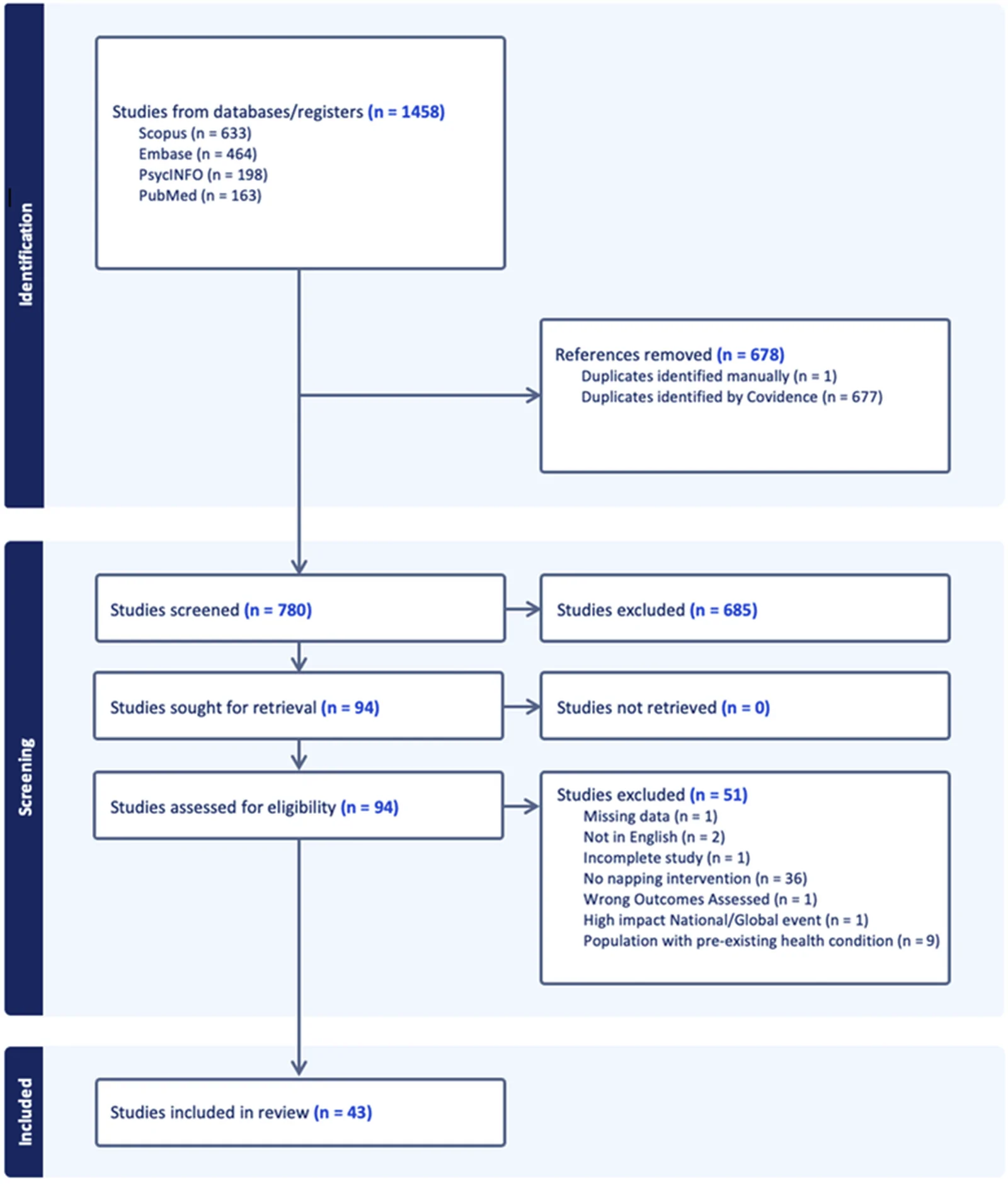
Prisma flow diagram of included studies.

The number of studies excluded at each stage and the reasons for exclusion are shown in Figure 1.

### Quality Appraisal

There were twenty-seven cross-sectional studies, fifteen prospective cohort studies, and 1 case-control study appraised for quality. Scores ranged from 38% to 91%. Cross-sectional studies showed the widest variability (38-88%) and were often limited by reliance on invalidated self-report nap measures and minimal adjustment for covariates. Many also focused on very elderly Mediterranean samples, such as the centenarian study by Spadafora et al,^30^ which was largely descriptive and received the lowest quality score (38%). In contrast, several other cross-sectional investigations, along with most prospective cohorts and the single case-control study, achieved higher scores by using validated outcome measures and accounting for confounds through more complex statistical models.

The overall quality assessment for each study is presented in Tables 1-3. Studies scoring <50% were considered low quality but were still included in the review to ensure all available data were considered and to reflect the full picture of existing research.

**Table 1.** Case-Control Quality Assessment Results.

Lead Author, Year	Q1	Q2	Q3	Q4	Q5	Q6	Q7	Q8	Q9	Q10	Total score
Turner, 2022	✓	✓	✓	X	✓	✓	✓	✓	X	✓	80%

**Table 2.** Cross-Sectional Quality Assessment Results.

Lead Author, Year	Q1	Q2	Q3	Q4	Q5	Q6	Q7	Q8	Total score
Panagiotakos 2011	✓	✓	X	✓	X	X	✓	✓	63%
Citoni, 2014	✓	✓	X	X	✓	✓	X	✓	63%
Cournot 2004	✓	✓	X	✓	✓	✓	✓	✓	88%
Ferranti 2016	✓	✓	X	✓	✓	✓	✓	✓	88%
Fidancı 2022	✓	✓	X	✓	✓	X	✓	✓	75%
Foscolou 2020	✓	✓	X	✓	✓	✓	✓	✓	88%
Fountoulakis 2022	✓	✓	X	X	✓	✓	X	✓	63%
Frisoni 1996	✓	✓	X	✓	✓	✓	✓	✓	88%
Georgiadi 2022	✓	✓	X	X	✓	✓	X	✓	63%
Georgousopoulou 2017a	✓	✓	X	✓	✓	✓	✓	✓	88%
Georgousopoulou 2017b	✓	✓	X	✓	✓	✓	✓	✓	88%
Hashem 2023	✓	✓	X	✓	✓	✓	✓	✓	88%
Katsa 2018	✓	✓	X	✓	✓	✓	✓	✓	88%
Léger 2019	✓	✓	X	X	✓	✓	X	✓	63%
Ltifi 2025	✓	✓	✓	✓	✓	X	✓	✓	88%
Malloggi 2020	✓	✓	X	X	✓	✓	X	✓	63%
Mesas 2022	✓	✓	X	✓	✓	X	✓	✓	75%
Ohayon 2005	✓	✓	X	✓	✓	✓	X	✓	75%
Orhan 2012	✓	✓	X	✓	X	X	✓	✓	63%
Özder 2015	✓	✓	X	✓	✓	✓	✓	✓	88%
Serdari 2020	✓	✓	X	✓	✓	✓	✓	✓	88%
Settineri 2012	✓	✓	X	X	✓	✓	X	✓	63%
Spadafora 1996	✓	✓	X	✓	X	X	X	X	38%
Tourlouki 2010	✓	✓	X	✓	X	X	✓	✓	63%
Tsiptsios 2021	✓	✓	X	✓	✓	✓	✓	✓	88%
Tsiptsios 2022	✓	✓	X	X	✓	✓	X	✓	63%
Vorvolakos 2020	✓	✓	X	✓	✓	✓	✓	✓	88%

**Table 3.** Prospective Cohort Quality Assessment Results.

Lead Author, Year	Q1	Q2	Q3	Q4	Q5	Q6	Q7	Q8	Q9	Q10	Q11	Total score
Bairami 2025	✓	✓	X	✓	✓	✓	✓	Unclear	✓	X	✓	73%
Barbería-Latasa 2023	✓	✓	X	✓	✓	✓	✓	✓	✓	X	✓	82%
Burazeri 2003a	✓	✓	X	✓	✓	✓	✓	✓	✓	X	✓	82%
Burazeri 2003b	✓	✓	X	✓	✓	✓	✓	✓	✓	X	✓	82%
Bursztyn 1999	✓	✓	X	✓	✓	✓	✓	✓	X	X	✓	73%
Cohen-Mansfield 2012	✓	✓	X	✓	✓	✓	✓	✓	✓	✓	✓	91%
Díaz-Gutiérrez 2018	✓	✓	X	✓	✓	✓	✓	✓	✓	X	✓	82%
Garralda-Del-Villar 2019	✓	✓	X	✓	✓	✓	X	✓	✓	X	✓	73%
Gribble 2021	✓	✓	X	✓	✓	✓	X	✓	✓	X	✓	73%
Moreno-Montañés 2022	✓	✓	X	✓	✓	✓	✓	✓	✓	X	✓	82%
Naska 2007	✓	✓	X	✓	✓	✓	✓	X	✓	X	✓	73%
Ruiz-Estigarribia 2019	✓	✓	X	✓	✓	✓	✓	✓	✓	X	✓	82%
Ruiz-Estigarribia 2020a	✓	✓	X	✓	✓	✓	✓	✓	✓	X	✓	82%
Ruiz-Estigarribia 2020b	✓	✓	X	✓	✓	✓	✓	✓	✓	✓	✓	91%
Sayón-Orea 2013	✓	✓	X	✓	✓	✓	✓	✓	✓	X	✓	82%

### Impacts of Naps

In total, forty-three studies investigated outcomes relating to health, quality of life, or lifespan, with 9 examining multiple domains.^31-39^ For clarity, studies were organised by outcome domain and summarised in Tables 4–12, which provide an overview of study characteristics, nap characteristics assessed, outcome measures, and key findings. The results are presented thematically across 9 domains: (a) lifespan (n = 10), (b) quality of life (n = 2), (c) mental health (n = 9), (d) hypertension and cardiovascular (n = 9), (e) BMI and metabolic (n = 14), (f) Sleep (n = 5), (g) cognitive (n = 2), (h) motor function (n = 3), and (i) Other outcomes (n = 3).

**Table 4.** Associations Between Napping and Lifespan and Mortality Outcomes.

Author, Year	Country & Population	Study Design	Sleep Disorders accounted for?	Daytime Sleepiness accounted for?	Nap Assessment & characteristics	Outcome(s) measured	Key findings	Napping impact
Barbería-Latasa et al, 2023	Spain; N = 17,747, F: 60.55%, Mean age: 37.2 ± 11.9 years, SUN cohort	Prospective cohort study	No. Sleep disorders were not screened, recorded, or controlled for.	No. Daytime sleepiness was not screened, recorded, or controlled for.	Self-report questionnaire. Duration: Siesta (no), short siesta (≤30min/day), and long siesta (>30min/day)	All-cause mortality: Total (any age); premature mortality (≤65 years); late mortality (>65 years)	Long naps were associated with increased total (*HR* = 1.95, 95% *CI* [1.29-2.94]) and late mortality risk (*HR* = 2.53, 95% *CI* [1.30-4.91]) compared to no naps.; no significant association for premature mortality or short naps.	−
Burazeri et al, 2003a	Israel; N = 1,842, F: 54.34%, Median age: 64 years, Kiryat Yovel community health study	Prospective cohort study	No. Sleep disorders were not screened, recorded, or controlled for. The authors acknowledged the absence of data on snoring and noted sleep apnoea as a possible unmeasured confounder.	No. Daytime sleepiness was not screened, recorded, or controlled for.	Self-report questionnaire; siesta (yes/no) duration: Average duration/day, long nap (≥2 hours)	All-cause and cause-specific (cardiovascular) mortality	Long total sleep duration was associated with higher mortality in men, but daytime napping did not attenuate this risk; no association in women.	±
Burazeri et al, 2003b	Israel; N = 1,842, F: 54.34%, Median age: 64 years, Kiryat Yovel community health study	Prospective cohort study	No. Sleep disorders were not screened, recorded, or controlled for. The authors acknowledged the absence of data on snoring and noted sleep apnoea as a possible unmeasured confounder.	No. Daytime sleepiness was not screened, recorded, or controlled for.	Self-report questionnaire; siesta (yes/no) duration: No siesta, <1hour, 1hour, ≥2 hours (long nap)	All-cause and cause-specific (cardiovascular) mortality	Long naps were associated with all-cause (*HR* = 2.01, 95% *CI* [1.24-3.27]) and cause-specific (*HR* = 2.60, 95% *CI* [1.30-5.21]) mortality in men; no association in women.	−
Bursztyn et al, 1999	Israel; N = 455, F: 38.4%, age: 70-year-olds	Prospective cohort study	No. Sleep disorders were not screened, recorded, or controlled for. The authors discussed obstructive sleep apnoea as a potential confounder but did not have data on it. Snoring was recorded but was not associated with mortality.	No. Daytime sleepiness was not screened, recorded, or controlled for.	Self-report questionnaire; siesta (yes/no)	All-cause Mortality	Napping was associated with higher mortality (*OR* = 2.1, 95% *CI* [1.1-3.9]); napping is an independent predictor of mortality.	−
Cohen-Mansfield & Perach, 2012	Israel; N = 1,166, age: ≥75 years Mean age: 83.4 ± 5.3, CALAS cohort	Prospective cohort study	No. Sleep disorders were not screened, recorded, or controlled for. The authors acknowledged this limitation and noted the absence of sleep assessment technology.	No. Daytime sleepiness was not screened, recorded, or controlled for.	Self-report questionnaire; daytime napping (yes/no/sometimes), duration: No nap, 1 hr, >2 hr)	All-cause Mortality	No direct association between daytime napping and mortality.	±
Naska et al, 2007	Greece; N = 23,681, F: NR, age range: 20-86 years, EPIC cohort	Prospective cohort study	No. Sleep disorders were not screened, recorded, or controlled for.	No. Daytime sleepiness was not screened, recorded, or controlled for. The study distinguished habitual siesta from daytime sleepiness conceptually but did not measure sleepiness.	Self-report questionnaire; siesta (yes/no), duration: The average duration of nap (in 5-minute increments), frequency: No naps, occasionally (<3 timer per week), systematically (≥3 times per week)	Coronary heart disease mortality	Naps of any duration or frequency was associated with lower coronary mortality (MR = 0.66, 95% *CI* [0.45-0.97]). Systematically napping had 37% lower coronary mortality (*MR* = 0.63, 95% *CI* [0.42-0.93]). No significant association for occasional napping.	+
Panagiotakos et al, 2011	Greece; N = 187, F: 52%, age: ≥80 years, Mean age: 84 ± 4 years, Ikaria Island study	Cross-sectional study	No. Sleep disorders were not screened, recorded, or controlled for.	No. Daytime sleepiness was not screened, recorded, or controlled for.	Self-report questionnaire; daily siesta (yes/no)	Lifespan	Majority of long-living participants reported daily naps, suggesting napping may be associated with longer lifespan.	+
Ruiz-Estigarribia et al, 2020a	Spain; N = 20,094, F: NR, age: NR, SUN cohort	Prospective cohort study	No. Sleep disorders were not screened, recorded, or controlled for.	No. Daytime sleepiness was not screened, recorded, or controlled for.	Self-report questionnaire; mid-day nap (yes/no), duration: hours per day, short mid-day nap (≤30min/day), long mid-day nap (>30min/day)	All-cause mortality	Short midday naps were associated with lower all-cause mortality (*HR* = 0.67, 95% *CI* = 0.54-0.83); longer naps showed no impact.	+
Spadafora et al, 1996	Italy; N = 48, F: 75.0%, age: ≥100 years, Mean age: 102 ± 1.9 years	Cross-sectional study	Yes, partially. A custom questionnaire recorded whether sleep was interrupted or uninterrupted during the night, and use of sleeping pills. No sleep disorder instrument was used.	No. Daytime sleepiness was not screened, recorded, or controlled for.	Self-report questionnaire; afternoon siesta (yes/no)	Lifespan	Afternoon napping was highly prevalent among centenarians (87.5%), suggesting a potential link between napping and longer lifespan.	+
Tourlouki et al, 2010	Greece & Cyprus; N = 1,190, F: 53.5%, age: ≥65	Cross-sectional study	No. Sleep disorders were not screened, recorded, or controlled for.	No. Daytime sleepiness was not screened, recorded, or controlled for.	Self-report questionnaire; regular mid-day nap (≥5 days/week; yes/no)	Lifespan	Majority of participants in all age groups (65-80, 80-90, >90) reported napping regularly. 100% of participants over 90 years reported regular mid-day naps suggesting a potential link between napping and longer lifespan.	+

*Note.* +, napping benefits; −, napping deficits; ±, no significant association; CALAS, Cross-Sectional and Longitudinal Aging Study; *CI,* confidence interval; EPIC, Greek European Prospective Investigation into Cancer and Nutrition cohort; F, female; *HR*, hazard ratio; MR, mortality ratio; NR, not reported; *OR*, odds ratio; SUN, Seguimiento Universidad de Navarra cohort. Statistics are included where reported in the source studies.

**Table 5.** Associations Between Napping and Quality of Life Outcomes.

Author, year	Country & population	Study design	Sleep Disorders accounted for?	Daytime sleepiness accounted for?	Nap assessment & characteristics	Outcome(s) measured (Instrument)	Key findings	Napping impact
Citoni, 2014	Italy; N = 15,550, F: 25.1%; age: ≥15 years, Median age: 45	Cross-sectional study	Partially. Insomnia was recorded as a variable from diaries. However, other sleep disorders (e.g. sleep apnoea) were not screened or recorded or controlled for.	No. Daytime sleepiness was not screened, recorded, or controlled for.	Self-report questionnaire; napping (yes/no)	Quality of life (HRQL)	Napping reduces quality of life by between 1% and 2% (*P* < 0.01)	−
Foscolou et al, 2020	Greece, Cyprus, Italy, Spain, Malta, Turkey; N = 2564; F: 46%; age: ≥65 years, MEDIS study	Cross-sectional study	No. Sleep disorders were not screened, recorded, or controlled for.	No. Daytime sleepiness was not screened, recorded, or controlled for.	Self-report questionnaire; Mid-day napper (≥5 days/week; yes/no)	Quality of life (SAI)	1. Midday nappers had 6.7% higher SAI score compared to non-nappers (*P* < 0.001) 2. The effect of midday napping was more prominent among males and participants ≥80.	+

*Note.* +, napping benefits; −, napping deficits; ±, no significant association; F, female; HRQL, Health Related Quality of Life; MEDIS, Mediterranean Islands Study; NR, not reported; SAI, Successful Ageing Index. Statistics are included where reported in the source studies.

**Table 6.** Associations Between Napping and Mental Health Outcomes.

Author, year	Country & population	Study design	Sleep disorders accounted for?	Daytime sleepiness accounted for?	Nap assessment & characteristics	Outcome(s) measured (Instrument)	Key findings	Napping impact
Panagiotakos et al, 2011	Greece; N = 187, F: 52%, age: ≥80 years, Mean age: 84±4 years, Ikaria Island study	Cross-sectional study	No. Sleep disorders were not screened, recorded, or controlled for.	No. Daytime sleepiness was not screened, recorded, or controlled for.	Self-report questionnaire; daily siesta (yes/no)	Depression (GDS)	Daily siesta was associated with lower GDS scores compared those who did not follow this habit (3.4 ± 3.0 vs 5.8±4.2, *P* < .001)	+
Fidancı et al, 2022	Turkey; N = 248; Case: N = 102; F: 68.1%, age range: 12-18 years Control: N = 110 F: 70%; Mean range: 12-18 years	Cross-sectional study	Yes. Partially. A 15-item sleep habits questionnaire assessed difficulty falling asleep, frequent waking, difficulty waking up, changes in sleep time, sleepwalking, nightmares, and snoring. No validated clinical sleep disorder instrument was used.	No. Daytime sleepiness was not screened, recorded, or controlled for.	Self-report questionnaire; daytime napping (yes/no)	Suicide attempts	Daytime napping was significantly more common among individuals with a history of suicide attempts *(P* = 0.012).	−
Frisoni et al, 1996	Italy; N = 223; F: 67.7%; age: ≥75 years Mean age: NR	Cross-sectional study	Partially. Four night sleep symptoms were assessed (difficulty falling asleep, waking frequently, early-morning awakenings, not feeling rested in the morning) via questionnaire. No validated sleep disorder instruments were used.	Daytime sleepiness was not screened, recorded, or controlled for.	Structured interview; Weekly nap frequency (days): None, 1-2, 3-4, ≥5.	Somatisation, depression, anxiety, paranoid ideation, psychoticism, OCD all assessed using the BSI.	1. OCD symptoms were significantly associated with napping ≥1 day/week (OR = 2.35, 99% CI [1.28-4.35] (note: CI derived by exponentiating B coefficient CI), *P* = .005). 2. Napping was not significantly associated with somatisation (*P* = .164), depression (*P* = .241), anxiety (*P* = .371), or paranoid ideation (*P* = .266).	−
Ohayon & Vecchierini, 2005	France; N = 1026; F: NR; age: ≥60 years	Cross-sectional study	Yes, the Sleep-EVAL system was used, which applies DSM-IV and ICSD diagnostic criteria. Insomnia (with and without excessive daytime sleepiness) and obstructive sleep apnoea syndrome were formally diagnosed and included in logistic regression models.	Yes. Daytime sleepiness was evaluated and used as a variable in analyses. Insomnia accompanied by excessive daytime sleepiness was distinguished from insomnia alone.	Computerised structured interview (Sleep-EVAL System); daytime sleep duration (min/day)	Psychological well-being (depression and anxiety; GWBS)	Psychological well-being (anxiety and depression) was not independently linked to daytime sleep.	±
Orhan et al, 2012	Turkey; N = 73; F: 37%; age: ≥60 years, Mean age: 74.0 years	Cross-sectional study	Yes, partially. Pittsburgh Sleep quality index (PSQI) was used to distinguish good (≤5) and poor (>5) sleepers. Sleep disorders were not screened, recorded, or controlled for.	Yes. Epworth sleepiness Scale (ESS) 19.2% participants had excessive daytime sleepiness ESS >10, but this was not used/controlled for in analyses.	Sleep diary; daytime nap frequency (nap/day) and duration (hour)	Depression (GDS)	Nap frequency (*r* = 0.365, *P* = 0.002) and nap duration (*r* = 0.280, *P* = 0.017) were positively correlated with depression	−
Léger et al, 2019	France; N = 57105, age ≥18 years	Cross-sectional study	No. Sleep disorders were not screened, recorded, or controlled for.	Yes. Daytime sleepiness was assessed using the Epworth Sleepiness Scale (ESS). Excessive daytime sleepiness was defined as ESS >10 and included as a covariate in the multivariable models.	Responded (Yes/No) for weekday or week-end napping.	Depression and anxiety	Individuals that had depression and anxiety, had a significantly increased likelihood of napping compared with individuals without these disorders. The adjusted ORs were from 1.24 and 1.19 respectively.	−
Ruiz-Estigarribia et al, 2019	Spain; N = 14,908, F: 59.5%; Mean age: 36.7±11.6 years, SUN cohort	Prospective cohort study	No. Sleep disorders were not screened, recorded, or controlled for.	No. Daytime sleepiness was not screened, recorded, or controlled for.	Self-report questionnaire; short mid-day nap: yes (0.1h-0.5h/day) or no (no nap or nap duration of >0.5h/day)	Depression (self-report of diagnosis)	No significant, independent association of siesta with incident of depression.	±
Serdari et al, 2020	Greece; N = 957, F: 54.1%; Mean age: 49.62±14.79 years, Thrace cohort	Cross-sectional study	Yes. Four validated instruments were used: Athens Insomnia Scale (AIS) for insomnia; Pittsburgh Sleep Quality Index (PSQI) for sleep quality; Berlin Questionnaire (BQ) for OSA risk. Use of sleep medication was also recorded. All were included in adjusted regression models.	Yes. The Epworth Sleepiness Scale (ESS) was administered. Excessive daytime sleepiness prevalence was 8.7%. ESS scores were associated with anxiety in adjusted models (aOR = 5.20).	Self-report questionnaire; daytime napping (yes/no)	Anxiety (Zung Self-Rating Anxiety Scale)	No independent association between daytime napping and anxiety (*P* = 0.466).	±
Vorvolakos et al, 2020	Greece; N = 957, F: 54.1%; Mean age: 49.62±14.79 years, Thrace cohort	Cross-sectional study	Yes, Athens Insomnia Scale (AIS), Pittsburgh Sleep quality index (PSQI), and Berlin Questionnaire (for obstructive sleep apnoea risk). Also assessed insomnia subtypes: Difficulty initiating sleep, difficulty maintaining sleep, and early-morning awakening.	Yes. Epworth Sleepiness Scale (ESS) was used. Prevalence of excessive daytime sleepiness was 8.7%. No significant association with depression was found.	Self-report questionnaire; daytime napping (yes/no)	Depression (BDI – Greek version)	No association between daytime napping and depressive symptoms (*P* = 0.878).	±

*Note.* +, napping benefits; −, napping deficits; ±, no significant association; BDI, Beck Depression Inventory; BSI, Brief Symptom Inventory; ESS, Epworth Sleepiness Scale; F, female; GDS, Geriatric Depression Scale; GWBS, General Well-Being Schedule; HADS, Hospital Anxiety and Depression Scale; NR, not reported; OCD, obsessive-compulsive disorder; *OR,* odds ratio. Statistics are included where reported in the source studies.

**Table 7.** Associations Between Napping and Hypertension and Cardiovascular Outcomes.

Author, year	Country & population	Study Design	Sleep disorders accounted for?	Daytime sleepiness accounted for?	Nap assessment & characteristics	Outcome(s) measured	Key findings	Napping impact
Díaz-Gutiérrez et al, 2018	Spain; N = 19,336, F: 61.5%, Mean age: NR, SUN cohort	Prospective cohort study	No. Sleep disorders were not screened, recorded, or controlled for.	No. Daytime sleepiness was not screened, recorded, or controlled for.	Self-report questionnaire; duration afternoon nap (min/day); short afternoon nap (≤30 min/day) vs no nap or long nap (>30 min/day)	CVD	Napping was associated with reduced risk of CVD events when naps were short (*HR* = 0.67, 95% *CI* [0.46-0.95]) compared to no nap or long naps	+
Foscolou et al, 2020	Greece, Cyprus, Italy, Spain, Malta, Turkey; N = 2564; F: 46%; age: ≥65 years, MEDIS study	Cross-sectional study	No. Sleep disorders were not screened, recorded, or controlled for.	No. Daytime sleepiness was not screened, recorded, or controlled for.	Self-report questionnaire; Mid-day napper (≥5 days/week; yes/no)	Hypertension	Napping was significantly associated with prevalence of hypertension (*P* = 0.002).	−
Fountoulakis et al, 2022	Greece; N = 957, F: 54.1%; Mean age: 49.62 ± 14.79 years, Thrace cohort	Cross-sectional study	Yes. Athens Insomnia Scale (AIS), Pittsburgh Sleep quality index (PSQI), and Berlin Questionnaire (BQ) for obstructive sleep apnoea risk. Sleep duration and efficiency were also calculated.	Yes. The Epworth Sleepiness Scale (ESS) was used to formally assess excessive daytime sleepiness (prevalence: 8.7%).	Self-report questionnaire; daytime napping (yes/no)	CVD	Napping was not associated with CVD prevalence (*P* = 0.533).	±
Georgousopoulou et al, 2017b	Greece, Cyprus, Italy, Spain, Malta, Turkey; N = 2749; F: 50.2%; age: ≥65 years, MEDIS study	Cross-sectional study	No. Sleep disorders were not screened, recorded, or controlled for.	No. Daytime sleepiness was not screened, recorded, or controlled for.	Self-report questionnaire; regular siesta (≥5 days/week; yes/no)	Hypertension	Napping was associated with higher odds of hypertension (OR = 2.07, 95% CI [1.07-4.02]).	−
Katsa et al, 2018	Greece; N = 480, F: NR, age range: 5-12 years	Cross-sectional study	No. Sleep disorders were not screened, recorded, or controlled for.	No. Daytime sleepiness was not screened, recorded, or controlled for.	Self-report questionnaire; midday nap (yes/no); midday nap duration (hr/day)	High blood pressure	Midday napping was negatively correlated with high blood pressure markers SBP (*P* = .001) and DBP (*P* = .046)	+
Mesas et al, 2022	Spain; N = 698; F: 56.2%; Mean age: 13.9 ± 1.2, age range: 12-17 years	Cross-sectional study	No. Sleep disorders were not screened, recorded, or controlled for.	No. Daytime sleepiness was not screened, recorded, or controlled for.	Self-report questionnaire; usual siesta (yes/no), duration: Ordinal variable from 0-120mins	Blood pressure (SBP, DBP)	Napping was not associated with SBP or DBP (*P* > .05).	±
Ohayon & Vecchierini, 2005	France; N = 1026; F: NR; age: ≥60 years	Cross-sectional study	Yes. Sleep-EVAL system used (applies DSM-IV and ICSD criteria). Insomnia (with and without excessive daytime sleepiness) and obstructive sleep apnoea syndrome were formally diagnosed and included in logistic regression models.	Yes. From Sleep-EVAL system. Used as a variable in analyses. Insomnia accompanied by excessive daytime sleepiness was distinguished from insomnia alone.	Computerised structured interview (Sleep-EVAL System); daytime sleep duration (min/day)	High blood pressure	Napping ≥1hr/day was associated with greater risk of high blood pressure *(OR* = 1.5, 95% *CI* [1.0-2.3])	**-**
Léger et al, 2019	France; N = 57105, age ≥18 years	Cross-sectional study	No. Sleep disorders were not screened, recorded, or controlled for.	Yes. Daytime sleepiness was assessed using the Epworth Sleepiness Scale (ESS). Excessive daytime sleepiness was defined as ESS >10 and included as a covariate in the multivariable models.	Responded (Yes/No) for weekday or week-end napping.	Hypertension, diabetes (type 1 & 2), CVD.	Individuals had hypertension, diabetes (type 1 and 2), had a significantly increased likelihood of napping compared with individuals without these disorders. The adjusted ORs were from 1.14 and 1.28. respectively. CVD was not significant.	−
Tsiptsios et al, 2021	Greece; N = 957, F: 54.1%; Mean age: 49.62 ± 14.79 years, Thrace cohort	Cross-sectional study	Yes: Athens insomnia Scale (AIS) for insomnia; Pittsburgh Sleep Quality Index (PSQI) for sleep quality; Berlin Questionnaire (BQ) for OSA risk. Use of sleep medication was also recorded. All were included in adjusted regression models.	Yes. Epworth Sleepiness Scale (ESS). Excessive daytime sleepiness was found in 8.7% of participants and was significantly associated with hypertension (aOR = 2.72).	Self-report questionnaire; duration afternoon nap (min/day); short afternoon nap (≤30 min/day) vs no nap or long nap (>30 min/day))	Hypertension	Not napping during the day was a statistically significant determinant of hypertension (a*OR* = 2.00, 95% *CI* [1.31-3.04], *P* = 0.001).	+

*Note.* +, napping benefits; −, napping deficits; ± , no significant association. *aOR,* adjusted odds ratio; *CI,* confidence interval; CVD, cardiovascular disease; DBP, diastolic blood pressure; F, female; *HR,* hazard ratio; MEDIS, Mediterranean Islands Study; NR, not reported; *OR*, odds ratio; SBP, systolic blood pressure; SUN, Seguimiento Universidad de Navarra cohort. Statistics are included where reported in the source studies.

**Table 8.** Associations Between Napping and BMI and Metabolic Outcomes

Author, year	Country & population	Study design	Sleep disorders accounted for?	Daytime sleepiness accounted for?	Nap assessment & characteristics	Outcome(s) measured	Key findings	Napping impact
Cournot et al, 2004	France; N = 3,127, F: 48.7%, age groups: 32, 42, 52, or 62 years	Cross-sectional study	No. Sleep disorders were not screened, recorded, or controlled for.	No. Daytime sleepiness was not screened, recorded, or controlled for.	Self-report questionnaire; nap (<once a week or ≥ once a week)	BMI	Napping once or more a week was associated with higher BMI in women *(P* < .01); no significant association in men.	**−**
Ferranti et al, 2016	Italy; N = 1,586, F: NR, age range: 11-14 years	Cross-sectional study	Partially. Pediatric Daytime Sleepiness Scale (PDSS), which captures aspects of sleep disturbance. No formal clinical sleep disorder diagnosis.	Yes. The PDSS was used to assess daytime sleepiness, with higher scores indicating greater sleepiness.	Self-report questionnaire; nap on weekdays (yes/no), nap on weekends (yes/no), and nap duration (min/day)	BMI	No napping habits or duration were associated with BMI.	±
Foscolou et al, 2020	Greece, Cyprus, Italy, Spain, Malta, Turkey; N = 2564; F: 46%; age: ≥65 years, MEDIS study	Cross-sectional study	No. Sleep disorders were not screened, recorded, or controlled for.	No. Daytime sleepiness was not screened, recorded, or controlled for.	Self-report questionnaire; Mid-day napper (≥5 days/week; yes/no)	BMI; T2DM; hypercholesterolaemia	Napping was not associated with BMI (*P* = 0.72), T2DM (*P* = 0.14), or hypercholesterolaemia (*P* = 0.28).	±
Garralda-Del-Villar et al, 2019	Spain; N = 10,807, F: 67%, Mean age: 37 years, SUN cohort	Prospective cohort study	No. Sleep disorders were not screened, recorded, or controlled for.	No. Daytime sleepiness was not screened, recorded, or controlled for.	Self-report questionnaire; short afternoon nap 0.1-0.5 hr/day vs no nap or long nap >0.5 hr/day	Metabolic syndrome	A short afternoon nap was associated with lower Metabolic syndrome (*OR* = 0.79, 95% *CI* [0.64-0.96]) compared to longer or no naps.	+
Georgousopoulou et al, 2017a	Greece, Cyprus, Italy, Spain, Malta, Turkey; N = 2,749, F: NR, age: ≥65 years, MEDIS study	Cross-sectional study	No. Sleep disorders were not screened, recorded, or controlled for.	No. Daytime sleepiness was not screened, recorded, or controlled for.	Self-report questionnaire; Mid-day napper (≥5 days/week; yes/no)	Metabolic syndrome	Napping was associated with higher odds of Metabolic syndrome overall (OR = 4.05, 95% *CI* [1.31-12.52]; *P* < 0.05) and in females (*OR* = 3.43, 95% *CI* [1.08-10.90]); no association in males.	**−**
Georgousopoulou et al, 2017b	Greece, Cyprus, Italy, Spain, Malta, Turkey; N = 2749; F: 50.2%; age: ≥65 years, MEDIS study	Cross-sectional study	No. Sleep disorders were not screened, recorded, or controlled for.	No. Daytime sleepiness was not screened, recorded, or controlled for.	Self-report questionnaire; regular siesta (≥5 days/week; yes/no)	T2DM; hypercholesterolaemia	Napping was not associated with T2DM (*P* > 0.05) or hypercholesterolaemia (OR = 0.81, 95% CI [0.42-1.57]).	±
Gribble et al, 2021	Spain; N = 9,161, F: 68%, Mean age: 36 years, SUN cohort	Prospective cohort study	Yes, accounted for as covariables. Prior history of insomnia and obstructive sleep apnoea (OSA) were self-reported at baseline and included as covariables in adjusted logistic regression models. Snoring (a proxy for sleep-disordered breathing) was recorded and adjusted for in analyses.	No. Daytime sleepiness was not screened, recorded, or controlled for.	Self-report questionnaire; siesta (yes/no); duration: Short ≤30 min/day, long >30 min/day	Metabolic syndrome; obesity; low HDL cholesterol; high triglycerides; Hyperglycaemia	Long naps were associated with higher odds of Metabolic syndrome (*OR* = 1.39, 95% *CI* [1.03-1.88]), obesity *(OR* = 1.15-1.32, 95% *CI* [1.01-1.32]), and high triglycerides (*OR* = 1.33, 95% *CI* [1.00-1.76]); no significant association for hyperglycaemia or short siesta.	**−**
Katsa et al, 2018	Greece; N = 480, F: NR, age range: 5-12 years	Cross-sectional study	No. Sleep disorders were not screened, recorded, or controlled for.	No. Daytime sleepiness was not screened, recorded, or controlled for.	Self-report questionnaire; midday nap (yes/no); midday nap duration (hr/day)	Predisposition for Metabolic syndrome (biomarkers)	Midday naps were positively correlated with metabolic syndrome in those with predisposition for metabolic syndrome.	**−**
Ltifi et al, 2025	Tunisia; N = 118, F: 60.2%, age range: 3-5 years	Cross-sectional study	No. Sleep disorders were not screened, recorded, or controlled for.	No. Daytime sleepiness was not screened, recorded, or controlled for.	Parent questionnaire and observation; post-lunch napping (yes/no); duration: Short ≤60 min/day or long >60 min/day	BMI	Napping was associated with higher BMI (*P* < .0001) compared to no-nap.	**−**
Ohayon & Vecchierini, 2005	France; N = 1026; F: NR; age: ≥60 years	Cross-sectional study	Yes. Sleep-EVAL system used (applies DSM-IV and ICSD criteria). Insomnia (with and without excessive daytime sleepiness) and obstructive sleep apnoea syndrome were formally diagnosed and included in logistic regression models.	Yes. From Sleep-EVAL system. Used as a variable in analyses. Insomnia accompanied by excessive daytime sleepiness was distinguished from insomnia alone.	Computerised structured interview (Sleep-EVAL System); daytime sleep duration (min/day)	Obesity	Napping >1 hr/day was associated with higher odds of obesity (*OR* = 1.8, 95% *CI* [1.1-3.0]).	**−**
Ruiz-Estigarribia et al, 2020b	Spain; N = 11,005, F: 43.1%, Mean age: 40.2±12 years, SUN cohort	Prospective cohort study	No. Sleep disorders were not screened, recorded, or controlled for.	No. Daytime sleepiness was not screened, recorded, or controlled for.	Self-report questionnaire; short mid-day nap (≤30min/day; yes/no)	T2DM	No significant association between short mid-day naps and T2DM risk compared to no nap or longer naps (>30min/day).	±
Sayón-Orea et al, 2013	Spain; N = 9,470, F: NR, Mean age: 39±12 years, SUN cohort	Prospective cohort study	Partially. Insomnia (yes/no) and regular snoring (yes/no) were self-reported and included as covariates in adjusted models. No formal screening for OSA or other specific sleep disorders.	No. Daytime sleepiness was not screened, recorded, or controlled for.	Self-report questionnaire; siesta (yes/no); duration (never; 30 min; >30–<60 min; >60 min)	Obesity	Napping 30 min/day was associated with lower risk of obesity (*HR* = 0.67, 95% *CI* [0.46-0.96]); longer naps showed no significant association.	+
Tsiptsios et al, 2022	Greece; N = 957, F: 54.1%, Mean age: 49.62±14.79 years, age range: 19-86 years	Cross-sectional study	Yes. Athens insomnia Scale (AIS) for insomnia; Pittsburgh Sleep Quality Index (PSQI) for sleep quality; Berlin Questionnaire (BQ) for OSA risk. Use of sleep medication was also recorded. Insomnia subtypes were assessed. All were included in adjusted regression models.	Yes. Epworth Sleepiness Scale (ESS). No significant association between excessive daytime sleepiness and dyslipidemia was found (aOR = 0.72, *P* = 0.212).	Self-report questionnaire; nap during the day (yes/no)	Dyslipidaemia	No association with napping and dyslipidaemia (*P* = .834).	±
Léger et al, 2019	France; N = 57105, age ≥18 years	Cross-sectional study	No. Sleep disorders were not screened, recorded, or controlled for.	Yes. Daytime sleepiness was assessed using the Epworth Sleepiness Scale (ESS). Excessive daytime sleepiness was defined as ESS >10 and included as a covariate in the multivariable models.	Responded (Yes/No) for weekday or week-end napping.	Overweight or obesity	Individuals that were overweight or had obesity, had a significantly increased likelihood of napping compared with individuals without these disorders. The adjusted OR was 1.26.	**−**

*Note.* +, napping benefits; **−**, napping deficits;±, no significant association; BMI, body mass index; CI, confidence interval; F, female; HDL, high-density lipoprotein; HR, hazard ratio; MEDIS, Mediterranean Islands Study; MetSyn, metabolic syndrome; NR, not reported; OR, odds ratio; SUN, Seguimiento Universidad de Navarra cohort; T2DM, type 2 diabetes mellitus. Statistics are included where reported in the source studies.

**Table 9.** Associations Between Napping and Sleep Health Outcomes.

Author, year	Country & population	Study design	Sleep disorders accounted for?	Daytime sleepiness accounted for?	Nap assessment & characteristics	Outcome(s) measured	Key findings	Napping impact
Georgiadi et al (2022)	Greece; N = 957, F: 54.1%, Mean age: 49.62±14.8 years, age range: 19-86 years, Thrace	Cross-sectional study	No. Sleep disorders were not screened, recorded, or controlled for.	No. Daytime sleepiness was not screened, recorded, or controlled for.	Self-report questionnaire; short afternoon nap (≤30 min/day) vs no nap or long nap (>30 min/day)	Sleep efficiency	Napping for >30 min during the day is associated with worse sleep efficiency (*P* = 0.047)	**−**
Hashem et al (2023)	Egypt; N = 564, F: 41.1%, age range: 6-14 years, school children	Cross-sectional study	Yes. The Children’s Sleep Habits Questionnaire–Abbreviated (CSHQ-A) was used to identify sleep disorders risk (score >30). Various sleep behaviours including bedtime resistance, sleep onset delay, night wakings, and parasomnias were reccorded.	No. Daytime sleepiness was not screened, recorded, or controlled for.	Self-report questionnaire; daytime sleep duration (minutes)	Sleep problems in children (CSHQ-A)	Children with sleep problems reported significantly longer daytime sleep duration than those without sleep problems (*P* < .05).	**−**
Malloggi et al (2020)	Italy; N = 1,430, F: 49%, age range: 6-14 years	Cross-sectional study	Yes, screened. The School Sleep Habits Survey included a Sleep–Wake Problems Behaviour Scale (SWP; 10 items) assessing sleep/wake-related problems (e.g. nightmares, difficulty falling asleep, falling asleep in class). No clinical diagnoses of specific sleep disorders were made.	Yes, assessed. A Sleepiness Scale (SLS; 9 items) was administered, evaluating ease of staying awake in different situations (e.g. in class, watching TV, reading). Global sleepiness scores were calculated and compared between good and bad sleepers.	Self-report questionnaire; categorical nap frequency (sometimes school days, sometimes weekends, none)	Sleep quality (self-report)	No significant differences in sleep quality were found between napping frequency groups.	±
Özder and Eker (2015)	Turkey; N = 252, F: 33.3%, Mean age: 39.90±9.99, academic physicians	Cross-sectional study	Partially. Insomnia was recorded (yes/no). Regular sleep aid use (sleeping pills) was documented. Snoring was not assessed. No formal screening for OSA or other specific sleep disorders.	Yes. Daytime sleepiness was assessed using the Epworth Sleepiness Scale (ESS). EDS was defined as ESS >10.	Self-report questionnaire; habitual napping (yes/no)	Daytime sleepiness (ESS)	Habitual napping was positively associated with higher ESS scores (*P* < .01).	**−**
Orhan et al, 2012	Turkey; N = 73; F: 37%; age: ≥60 years, Mean age: 74.0 years	Cross-sectional study	Yes, screened. The Pittsburgh Sleep Quality Index (PSQI) was used to distinguish good (≤5) and poor (>5) sleepers. Sleep disturbances, sleep latency, sleep duration, and habitual sleep efficiency were assessed as PSQI subscales. A sleep diary was also kept for 14 days. No formal clinical sleep disorder diagnoses were made.	Yes, assessed. The Epworth Sleepiness Scale (ESS) was administered. Excessive daytime sleepiness was defined as ESS >10; 19.2% of participants met this criterion.	Sleep diary; daytime nap frequency (nap/day) and duration (hour)	Daytime sleepiness (ESS)	No significant association between nap frequency or duration and daytime sleepiness.	±

*Note.* +, napping benefits; −, napping deficits; ±, no significant association; F, female; CSHQ-A = Children’s Sleep Habits Questionnaire, abbreviated; ESS **=** Epworth Sleepiness Scale**;** EDS = excessive daytime sleepiness; NR = not reported. Statistics are included where reported in the source studies.

**Table 10.** Associations Between Napping and Cognitive Health Outcomes.

Author, year	Country & population	Study design	Sleep disorders accounted for?	Daytime sleepiness accounted for?	Nap assessment & characteristics	Outcome(s) measured	Key findings	Napping impact
Bairami et al, 2025	Greece; N = 955, F: NR; age ≥65 years, HELIAD cohort	Prospective cohort study	Partially. Sleep problems were assessed using the Sleep Index II from the Medical Outcomes Study Sleep Scale. This captured sleep disturbance, though specific clinical sleep disorders (e.g. sleep apnoea) were not formally diagnosed or screened.	Yes. Daytime somnolence was assessed using the somnolence subscale of the MOS Sleep Scale (items 6, 9, and 11) and was included as a predictor variable in Cox regression analyses.	Self-report questionnaire; nap frequency (ordinal: None, a little, some, a good bit, most, all)	MCI or dementia (neuropsychological assessment and MMSE)	One unit increase in nap frequency was associated with 19.3% lower risk of developing MCI or dementia (*HR* = 0.87, 95% CI [0.711-0.916]).	+
Ohayon & Vecchierini, 2005	France; N = 1026; F: NR; age: ≥60 years	Cross-sectional study	Yes. Sleep-EVAL system was used and obstructive sleep apnoea syndrome were formally diagnosed and included in logistic regression models.	Yes, assessed. Daytime sleepiness was evaluated and used as a variable in analyses. Insomnia accompanied by excessive daytime sleepiness was distinguished from insomnia alone.	Computerised structured interview (Sleep-EVAL System); daytime sleep duration (min/day)	Cognitive impairment (CDS & MMSE)	Napping >1hr/day was associated with higher prevalence of cognitive impairment (*OR* = 1.7, 95% *CI* [1.1-2.6]).	**−**

*Note.* +, napping benefits; −, napping deficits; ±, no significant association; CDS, Cognitive Difficulties Scale; F, female; HELIAD, Hellenic Longitudinal Investigation of Aging and Diet; *HR*, hazard ratio; MCI, mild cognitive impairment; MMSE, Mini-Mental State Examination; NR, not reported. Statistics are included where reported in the source studies.

**Table 11.** Associations Between Napping and Motor Function Outcomes.

Author, year	Country & population	Study design	Sleep disorders accounted for?	Daytime sleepiness accounted for?	Nap assessment & characteristics	Outcome(s) measured	Key findings	Napping impact
Frisoni et al, 1996	Italy; N = 223; F: 67.7%; age: ≥75 years Mean age: NR	Cross-sectional study	Partially. Four night sleep symptoms were assessed (difficulty falling asleep, waking frequently, early-morning awakenings, not feeling rested in the morning) via questionnaire. No validated sleep disorder instruments were used.	No. Daytime sleepiness was not screened, recorded, or controlled for.	Structured interview; Weekly nap frequency (days): None, 1-2, 3-4, ≥5.	Functional ability (BADL and IADL)	Napping was not significantly associated with functional ability in the BADL and IADL when adjusted for confounders (*P* > 0.05).	±
Ltifi et al, 2025	Tunisia; N = 118, F: 60.2%, age range: 3-5 years	Cross-sectional study	No. Sleep disorders were not screened, recorded, or controlled for.	No. Daytime sleepiness was not screened, recorded, or controlled for.	Parent questionnaire and observation; post-lunch napping (yes/no); duration: Short ≤60 min/day or long >60 min/day	Motor function (functional tasks assessed gross and fine motor skills)	Napping was associated with significantly better gross and fine motor performance among boys (*P* = 0.001); non-significant in girls (*P* = 0.977).	v
Ohayon & Vecchierini, 2005	France; N = 1026; F: NR; age: ≥60 years	Cross-sectional study	Yes. Sleep-EVAL system used (applies DSM-IV and ICSD criteria). Insomnia (with and without excessive daytime sleepiness) and obstructive sleep apnoea syndrome were formally diagnosed and included in logistic regression models.	Yes, using Sleep-EVAL system. Daytime sleepiness was evaluated and used as a variable in analyses. Insomnia accompanied by excessive daytime sleepiness was distinguished from insomnia alone.	Computerised structured interview (Sleep-EVAL System); daytime sleep duration (min/day)	Motor function (IADL)	Napping of any duration was not significantly associated with IADL scores.	±

*Note.* +, napping benefits; −, napping deficits; ±, no significant association; BADL, Basic Activities of Daily Living; F, female; IADL, Instrumental Activities of Daily Living; NR, not reported. Statistics are included where reported in the source studies.

**Table 12.** Associations Between Napping and Other Health Outcomes.

Author, year	Country & population	Study design	Sleep disorders accounted for?	Daytime sleepiness accounted for?	Nap assessment & characteristics	Outcome(s) measured	Key findings	Napping impact
Moreno-Montañés et al, 2022	Spain; N = 18,420, F: NR, age: NR, SUN cohort	Prospective cohort study	No. Sleep disorders were not screened, recorded, or controlled for.	No. Daytime sleepiness was not screened, recorded, or controlled for.	Self-report questionnaire; afternoon nap: yes (≤30 min/day) or no (no nap or nap duration of >30min/day)	Glaucoma	Napping was not independently associated with glaucoma incidence.	±
Settineri et al, 2012	Italy; N = 2,005, F: 31%, Mean age: 19 years, age range: 16-22 years	Cross-sectional study	Partially. A 12-item questionnaire assessed sleep disturbances including time to fall asleep, total sleep time, night awakenings, well-being upon awakening. These were used as predictors in a bivariate probit model. No validated sleep disorder screening instrument was used.	Yes. Item 12 specifically assessed daytime drowsiness, measuring both its occurrence and intensity on a 0-10 scale. It was also used as a dependent variable in the econometric model.	Self-report questionnaire; siesta (yes/no), nap duration (mins)	Joint incidence of depression and daytime drowsiness	Siesta was significantly associated with a small increase in the joint probability of depression and daytime drowsiness (marginal effect 0.03%).	**−**
Turner et al, 2022	Spain; N = 1581 breast cancer cases & 1609 controls; 1013 prostate cancer cases & 1179 controls; F: 59%; age range: 20-85 years	Case-control study	Partially. Self-reported data were collected on sleep duration, sleep problems (type, duration, timing), timing of sleep, frequent changes in sleep schedule, level of rest during sleep, and nocturnal awakenings. No validated sleep disorder instrument was used; no data on sleep apnoea were available.	No. Daytime sleepiness was not screened, recorded, or controlled for.	Self-report questionnaire; daytime napping(yes/no); duration: Average nap length (min); nap frequency (naps/week)	Breast cancer; prostate cancer	In women, napping was positively associated with breast cancer risk (*OR* = 1.20, 95% *CI* [1.02-1.42]) which strengthened with greater weekly nap frequency (*P* = 0.004) and nap duration (*P* = 0.001); no significant association between napping and prostate cancer in men.	**−**
Léger et al, 2019	France; N = 57105, age ≥18 years	Cross-sectional study	No. Sleep disorders were not screened, recorded, or controlled for.	Yes. Daytime sleepiness was assessed using the Epworth Sleepiness Scale (ESS). Excessive daytime sleepiness was defined as ESS >10 and included as a covariate in the multivariable models.	Responded (Yes/No) for weekday or week-end napping.	Breast cancer and prostrate cancer	Individuals that had breast cancer and prostrate cancer, showed no statistically significantly difference in likelihood of napping compared with individuals without these disorders.	±

*Note.* +, napping benefits; −, napping deficits; ±, no significant association; *CI*, confidence interval; F, female; NR, not reported; *OR*, odds ratio; SUN, Seguimiento Universidad de Navarra cohort. Statistics are included where reported in the source studies.

#### Lifespan

Ten studies examined the association between napping and lifespan or mortality: 5 (50%) reported benefits, 2 (20%) no association, and 3 (30%) poorer outcomes (Table 4). Only 1 study,^30^ considered nighttime sleep disruption as a factor related to napping. Most studies used a prospective cohort design (70%), and 3 used a cross-sectional design. No studies assessed or screened for sleep disorders or daytime sleepiness. Evidence across studies showed no consistent association between napping and lifespan. Short or systematic naps were generally associated with neutral or beneficial outcomes, whereas long naps in older men were more frequently associated with increased mortality risk. Benefits were reported from prospective studies for short naps^40^ and for regular napping regardless of duration.^41^ Cross-sectional studies similarly found that daily napping was widespread among long-living adults aged ≥80-100 years across Mediterranean samples.^30,39,42^ In contrast, poorer outcomes were concentrated in older participants, particularly men, with napping linked to higher mortality at age 70,^43^ greater late mortality risk in adults >65 years,^44^ and elevated all-cause and cardiovascular mortality in men only.^45^ Finally, null findings were reported when nap characteristics were defined broadly, with no distinction between short vs long naps or criteria for regularity.^46,47^

#### Quality of Life

Two cross-sectional studies examined the association between napping and quality of life, with 1 reporting benefits and 1 reporting poorer outcomes (Table 5). Citoni^48^ recorded self-reported insomnia as a variable from diaries. However, other sleep disorders (e.g. sleep apnoea) and daytime sleepiness were not screened or recorded or controlled for. Overall, findings were mixed. In older adults, regular midday napping (≥5 days/week) was associated with a 6.7% higher Successful Ageing Index, particularly among men and those ≥80 years.^31^ In contrast, napping was linked to small but statistically significant reductions (1-2%) in health-related quality of life,^48^ but the author notes the large sample size (n = 15,550) may have driven the statistical significance of this modest effect.

#### Mental Health

Nine studies examined the association between napping and mental health: 1 (11.1%) reported benefits, 4 (44.4%) no association, and 4 (44.4%) negative effects (Table 6). Three studies did not assess or screen for sleep disorders. Four studies did not assess or screen for daytime sleepiness. Findings are mostly from cross-sectional design and are inconsistent, with variability observed between age groups and nap characteristics. Only 1 study used a prospective cohort design. In older adults, daily siesta was linked to lower depression.^39^ Other studies reported higher obsessive-compulsive symptoms with more frequent napping^32^ and positive correlations between nap frequency, duration, and depression.^38^ Additionally, in a large French adult sample, individuals with depression and anxiety had a significantly increased likelihood of napping.^35^ In adolescents, daytime napping was more commonly reported in those with a history of suicide attempt.^49^ In other cross-sectional studies, napping was not associated with anxiety, depression, or psychological well-being.^37,50,51^ Evidence from a prospective study also showed no association between napping and depressive symptoms.^52^

#### Hypertension and Cardiovascular Outcomes

Nine studies (1 prospective, 8 cross-sectional) examined the association between napping and cardiovascular outcomes or hypertension: 3 (33.3%) reported benefits, 2 (22.2%) no association, and 4 (44.4%) negative effects (Table 7). Only 3 studies accounted for sleep disorders, and 4 accounted for daytime sleepiness in the analyses. Findings were mixed, with short naps appearing protective against cardiovascular disease in some studies, whereas longer or habitual napping was more frequently associated with higher blood pressure and hypertension, particularly in older adults.

Benefits were reported from a prospective study in which short afternoon naps (≤30 min/day) were associated with reduced cardiovascular risk compared with no naps or longer naps.^53^ Additionally, not napping during the day was a statistically significant determinant of hypertension in older Greek adults.^54^ Additionally, in Greek children (5-12 years old), midday naps were negatively correlated with both systolic and diastolic blood pressure.^34^ In contrast, poorer outcomes emerged in 4 studies, where napping was associated with higher odds of hypertension^31,33^ and a greater risk of high blood pressure with naps ≥1 hr/day in older adults.^37^ Additionally, in a large study of French adults, individuals with hypertension had a significantly increased likelihood of napping compared to those without hypertension.^35^ Finally, 2 studies identified no association, whereas 1 found no link between napping and cardiovascular disease prevalence,^55^ and the other reported no relationship with blood pressure in adolescents.^56^

#### Obesity and Metabolic Outcomes

Fourteen studies examined the association between napping and obesity (BMI) or metabolic outcomes: 2 prospective cohort studies (14.3%) reported benefits, 1 prospective cohort study and 6 cross-sectional studies (50.0%) reported poorer outcomes, and 4 cross-sectional studies and 1 prospective cohort study (35.7%) found no association (Table 8). Only 5 studies took into account or partially considered sleep disorders. Only 3 studies recorded and considered daytime sleepiness in their subsequent analyses. Evidence across studies showed no consistent pattern, although frequent or long naps were more often linked to increased risk of metabolic syndrome.

Individuals who reported for short naps (≤30 min/day) had reduced odds of metabolic syndrome compared to the ones who reported longer or no naps.^57^ Additionally, short nappers (≤30 min/day) showed a lower risk of obesity compared to non-nappers.^58^ In contrast, long naps (>30 min/day) were associated with higher odds of metabolic syndrome, obesity, and elevated triglycerides,^59^ and with obesity in older adults when naps exceeded 1 h/day.^37^ Cross-sectional studies, that did not document nap length, indicated higher metabolic risk among women who napped once or more per week,^60^ children predisposed to metabolic syndrome who took midday naps,^34^ and older adults where regular midday naps were associated with increased metabolic syndrome, particularly among women.^61^ Napping (with duration not indicated) was also associated with higher BMI in preschool children,^36^ and individuals who were overweight or had obesity had a significantly increased likelihood of napping.^35^ Finally, null findings were observed between napping and BMI in adolescents.^62^ Similarly, no significant associations were reported for regular napping (≥5 days/week) in relation to BMI, type 2 diabetes, or hypercholesterolaemia in older adults.^31,33^ No significant associations were also observed for short naps with type 2 diabetes^63^ or for napping with dyslipidaemia.^64^

#### Sleep Health

Five cross-sectional studies examined the association between napping and sleep health: 3 (60%) reported poorer outcomes and 2 (40%) reported no association (Table 9). One study did not assess or screen for sleep disorders, and 2 studies did not assess or screen for daytime sleepiness. Evidence across studies showed no consistent association between napping and sleep outcomes, with variability observed between age groups and nap frequency.

Long naps (>30 min/day) were associated with reduced sleep efficiency,^65^ while Egyptian school children with sleep problems reported significantly longer daytime sleep,^66^ and habitual napping was linked with greater daytime sleepiness among academic physicians.^67^ By contrast, null findings were observed in children, where nap frequency showed no association with sleep quality,^68^ and in older adults, where nap duration or frequency was unrelated to daytime sleepiness.^38^

#### Cognitive Health

One prospective cohort study and 1 cross-sectional study examined the association between napping and cognitive health. Benefits were reported in the prospective cohort of older Greek adults, where each unit increase in nap frequency was associated with a 19.3% lower risk of developing mild cognitive impairment or dementia.^69^ In contrast, poorer outcomes were observed in a cross-sectional study of French adults, where long naps (>1 hr/day) were associated with higher prevalence of cognitive impairment.^37^

#### Motor Functions

Three cross-sectional studies examined the association between napping and motor functions: 1 (33.3%) reported benefits, and 2 (66.7%) found no association (Table 11). One study did not assess or screen for sleep disorders, and 2 studies did not assess or screen for daytime sleepiness. Evidence was limited and inconsistent, with benefits observed in preschool-aged children but no significant effects reported in older adult populations.

Benefits were identified in a cross-sectional study of Tunisian preschool children, where post-lunch naps were associated with significantly better gross and fine motor performance among boys, although no effect was found in girls.^36^ In contrast, 2 cross-sectional studies of older adults reported null findings. Weekly nap frequency was not associated with basic or instrumental activities of daily living among Italians aged ≥75 years,^32^ and daytime napping of any duration showed no association with instrumental activities of daily living in French participants aged ≥60 years.^37^

#### Other Health Outcomes

Four studies (3 cross-sectional, 1 prospective cohort) examined the association between napping and other health outcomes: 2 (50%) reported no association, and 2 reported poorer outcomes (Table 12). Two studies partially accounted for sleep disorders by considering self-reported quality of nighttime sleep. Two studies assessed and accounted for daytime sleepiness in subsequent analyses. No associations were observed in a large Spanish cohort, where afternoon napping was unrelated to glaucoma incidence.^70^ In contrast, a case-control study found that daytime napping was associated with increased risk of breast cancer in women, with risk strengthening alongside greater nap frequency and duration, although no association was reported for prostate cancer in men.^71^ Similarly, a cross-sectional study of Italian young adults reported that napping was associated with a small increase in the joint probability of depression and daytime drowsiness in a recursive bivariate probit model analysis.^72^

## Discussion

This systematic review is the first to synthesise evidence on the impact of daytime napping across health outcomes, quality of life, and lifespan in Mediterranean populations, where napping is culturally embedded in daily routines. The review explored whether nap characteristics such as duration and frequency of naps influence outcomes, extending previous research by situating them in a region where napping is part of daily life. The findings reveal inconsistent results on the associations between napping and lifespan, quality of life, and health outcome domains. Evidence from 1 prospective cohort study^53^ suggested that short naps (≤30 min/day) may confer protective effects for cardiovascular disease. Furthermore, not napping was a statistically significant determinant of hypertension in a large cross-sectional study in Greek adults.^54^ However, studies investigating napping outcomes were complicated by methodological heterogeneity, including varied definitions of naps, timing, duration, and frequency, as well as a lack of account of sleep disorders and daytime sleepiness. Furthermore, this research was dominated by cross-sectional studies where causality cannot be determined.

### Nap Classification

A key finding of this review is the considerable inconsistency in how nap characteristics were defined and analysed, especially nap duration, complicating interpretation across studies. Short naps were usually defined as ≤30 minutes,^40^ whereas long naps ranged from >30 minutes,^44^ >60 minutes,^37^ or ≥2 hours.^45^ Such variability means a participant can be categorised differently across studies, making interpretation difficult. This distinction is important because experimental research suggests naps ≤30 minutes improve alertness without sleep inertia, whereas longer naps may disrupt circadian regulation and thus result in more adverse outcomes.^73^ Additionally, several analyses grouped long nappers together with non-nappers, obscuring differences between these distinct groups. Evidence from the SUN cohort shows that short naps reduced the risk of metabolic syndrome when compared with a combined group of long nappers but not non-nappers.^57,59^ Long naps often signal underlying illness, while not napping may reflect higher alertness.^35^ Frequency of napping also appeared important, though findings were inconsistent and often confounded by how nap duration was considered. Some studies found that frequent napping was associated with successful ageing, poorer metabolic outcomes, and mental health indicators.^32,42,60,61^ However, studies varied widely, with differences in study design, with napping recorded very differently across studies. This is important because existing literature consistently highlights nap regularity as a key determinant of cognitive, metabolic health, and lifespan outcomes.^2,74^ Without a comprehensive and standardised nap assessment, it is difficult to establish whether nap frequency reflects a beneficial or detrimental habit. Importantly, where frequency was not accounted for, both short naps (≤30 min/day) were protective against all-cause mortality^40^ and regular napping of any duration (≤3 naps/week) was linked with reduced mortality.^41^ These results suggest nap duration alone is insufficient to explain outcomes, and future research should integrate frequency and duration to clarify their impact on health. Additionally, some studies assessed afternoon siesta,^30,42^ while others measure any daytime sleep without specifying time of day.^46^ The timing of naps is likely to be highly relevant. Afternoon naps taken after the traditional midday meal align with the circadian dip in alertness which may make them restorative,^75^ whereas morning or late-evening naps interact differently with circadian rhythms, potentially delaying sleep onset and disrupting nocturnal sleep.^76^ This distinction is rarely considered yet may also underpin divergent health associations.

### Pre-existing Sleep Disorders and Excessive Daytime Sleepiness

Of the 43 included studies in this systematic review, only 17 (40%) screened, recorded, or accounted for sleep disorders. Five studies partially accounted for sleep disorders by documenting participants’ sleep quality. Furthermore, only 12 (28%) studies screened, recorded, or accounted for daytime sleepiness. With little evaluation or adjustment for sleep disorders or daytime sleepiness across most studies, and sleep disorders and excessive daytime sleepiness being a main driver behind the need to nap, this is a major unaccounted for confound between napping and health outcomes. Thus, it is very possible that undiagnosed sleep disorders such as OSA, remain in many of the included samples, especially among older adults and adults with obesity.

### Age and Nocturnal Sleep

The influence of age and nocturnal sleep patterns appears central to understanding the inconsistent associations between napping and health. Napping was linked to higher blood pressure and mortality in older adults,^37,45^ but to no association or benefits in younger populations.^34^ Conversely, opposite trends emerged for mental health and quality of life where napping was associated with benefits in older adults^31,39^ but with negative associations in younger groups.^48,72^ These findings suggest that nap impacts are not uniform across the life time but instead vary depending on the interaction between age-related changes in sleep and underlying health. In younger, healthier populations, napping may be a response to developmental and physiological changes^77^ or irregular schedules, insufficient nocturnal sleep, or psychosocial stress.^78^ In older populations, deteriorating health may influence napping as a compensatory strategy to counter fragmented night sleep and to restore daytime functioning.

Crucially, nap impacts also depend on nocturnal sleep duration, which is itself influenced by age.^3^ In a prospective cohort study, napping combined with long nocturnal sleep increased mortality risk,^46^ while a U-shaped association with psychological health^31^ corroborated evidence that both short and long 24-hour sleep times are linked to adverse outcomes.^79^ Napping with short nocturnal sleep was harmful before the age of 84 but potentially protective thereafter.^46^ This aligns with evidence that older adults experience greater difficulty falling and staying asleep.^80^ These factors help explain why nap habits increase as nocturnal sleep decreases with age and may account for the stronger links between napping and positive outcomes in older populations.

Taken together, the evidence suggests that the health impact of naps cannot be understood without considering both age and total sleep context. Future studies should therefore examine napping as part of the 24-hour sleep–wake cycle and across the ageing trajectory, rather than treating it as an isolated behaviour. More prospective studies are needed to determine whether the relationship between longer sleep duration and chronic disease reflects a true causal risk factor or merely an association driven by underlying conditions.

### Napping Contexts

Across Mediterranean populations, napping often showed no independent effects on specific health outcomes.^32,62,68^ For instance, napping was not directly associated with glaucoma incidence, yet when considered alongside other lifestyle behaviours such as diet and activity, it contributed to reduced risk.^70^ This aligns with evidence that naps may exert their effects most meaningfully when situated within a constellation of protective lifestyle behaviours.^4^ Within this context, naps are not merely physiological responses, but intentional, lifestyle-oriented practices embedded in broader routines of diet, rest, and sociality^6^ that collectively improve health.^81^ Recognising this integration underscores the importance of nap motivation. Planned naps that align with cultural rhythms may function as health-promoting behaviours, while compensatory naps taken in response to poor nocturnal sleep, illness, or fatigue often signal deteriorating health.^35^ Incorporating nap motivation into study designs would therefore provide a more nuanced understanding of when naps act as a culturally embedded, health-promoting practice, and when they instead signal declining health.

### Significance and Limitations

This review is the first to systematically evaluate napping in Mediterranean populations, where naps are culturally embedded within daily routines. Previous reviews have largely focused on international or non-specific populations and consistently linked habitual long naps with higher risk of metabolic syndrome, cardiovascular disease, and mortality.^5,16^ By focussing on the Mediterranean context, this review addresses a key gap in understanding whether health impacts differ when naps are mainly practiced as lifestyle habits rather than compensatory behaviours. Furthermore, documenting variability in nap classification and context helps explain inconsistent findings in Mediterranean populations and underscores the need for more rigorous, standardised research.

Despite its significance, this review has several limitations. Overall study quality was assessed as moderate, yet many studies failed to provide detailed information on nap frequency or duration, inconsistently reported timing, and relied on unvalidated self-report questions about napping, which are often single items about whether the person naps and/or how often they nap, without capturing detailed characteristics such as nap duration, timing and excessive daytime sleepiness. Only 17 (40%) studies screened, recorded, or accounted for sleep disorders and only 12 (28%) studies screened, recorded, or accounted for daytime sleepiness in the samples. Lower-quality studies were often cross-sectional and descriptive with limited adjustment for confounders, while even prospective cohorts cannot fully exclude reverse causation, as subclinical illness may influence nap frequency and duration. The interpretation is also limited by the repeated use of large cohorts such as SUN and MEDIS cohorts. While these datasets provide substantial statistical power, their overrepresentation risks inflating significance and limits generalisability across diverse Mediterranean populations.

Further methodological limitations include restricting inclusion to English-language studies, which may have introduced language bias by excluding evidence reported in Spanish, Italian, Greek, or Arabic. Heterogeneity in nap definitions, measurements, and outcomes also prevented meta-analysis, requiring reliance on narrative synthesis that is more vulnerable to interpretive bias. Finally, it is worthwhile highlighting that categorising the Mediterranean as a homogeneous group significantly overlooks the cultural variation across regions, occupations, and generations, which can mask important within-country differences in nap-health associations.

### Implications and Future Research

This review has several implications. Theoretically, it provides a culturally informed contribution to public health by showing that daytime naps can be understood as part of the Mediterranean lifestyle, alongside diet, activity, and social routines, rather than in isolation. This perspective moves beyond treating naps as isolated risk factors and instead recognises them as culturally embedded health behaviours shaped by multiple determinants, with both protective and adverse pathways. This suggests that future public health guidance should consider the role of naps within broader lifestyle contexts rather than as isolated behaviours. The review also advances theoretical understanding by suggesting naps may function less as direct causal agents and more as markers of underlying health, with long naps often reflecting preclinical illness or fatigue rather than inherent harm. Methodologically, the review identified the need for greater standardisation in nap research to accurately determine its role in health outcomes.

Future research should employ rigorous, standardised measures of napping that integrate validated questionnaires with objective tools such as actigraphy or mobile health applications to accurately capture nap duration, timing, and frequency alongside nighttime sleep. Clear distinctions are also required between planned naps taken for restoration and reactive naps driven by illness, fatigue, or sleep deprivation. This approach will allow investigators to examine napping in the context of nocturnal sleep duration and quality in combination, and to assess how they interact with age and other factors to shape napping behaviours and health outcomes. Finally, research in Mediterranean populations should situate naps within their broader cultural and lifestyle context, assessing interactions with diet, activity, and social rhythms. These refinements will be crucial in clarifying whether naps exert independent benefits or risks, or whether they primarily reflect underlying health status and cultural practices.

While this review focuses on the Mediterranean region, the current literature is skewed towards Southern European cohorts. Future research should prioritise underrepresented populations in North Africa and the Middle East (e.g. Tunisia, Egypt, and Lebanon) to provide a more culturally and geographically diverse understanding of napping practices across the entire Mediterranean basin.

## Conclusion

In summary, this systematic review found no consistent evidence that napping independently influences health outcomes, quality of life, or mortality in Mediterranean populations. While short naps occasionally appeared beneficial and long naps detrimental, results were inconsistent and shaped by methodological and health-related factors. Collectively, the findings suggest that napping is best understood not as a stand-alone determinant of health but as part of a broader lifestyle with specific focus on nocturnal sleep. Future research using standardised and methodologically rigorous approaches will be essential to determine whether napping plays a causal role in promoting health or primarily reflects underlying health status within Mediterranean populations.

## Supplemental Material

Supplemental Material - Rest Unassured: A Systematic Review on the Impacts of Daytime Napping in the Mediterranean on Health Outcomes, Quality of Life, and Lifespan Supplemental Material for Rest Unassured: A Systematic Review on the Impacts of Daytime Napping in the Mediterranean on Health Outcomes, Quality of Life, and Lifespan by L. Ellis, B. C. Barata, R. Conduit, B. de Courten, C. Itsiopoulos, G. A. G. A. Kennedy, O. Kolokotroni, R. Lawson, M. C. Mosquera, E. Aprilakis, A. Pavlou, S. Xenos, Y. Yap, L. S. Sidossis in American Journal of Lifestyle Medicine
