# Supplementary material for: Rest Unassured: A Systematic Review on the Impacts of Daytime Napping in the Mediterranean on Health Outcomes, Quality of Life, and Lifespan
Source: Am J Lifestyle Med. 2026 Sep 11:15598276261481759. Online ahead of print. doi: 10.1177/15598276261481759 (PMC13570968; doi:10.1177/15598276261481759)
Supplement: Supplementary file 1 [file sj-pdf-1-ajl-10.1177_15598276261481759.pdf]

Supplementary 1 – Key Search Terms

(mediterranean OR albania OR "Bosnia and Herzegovina" OR gibraltar OR algeria OR morocco OR france OR croatia OR montenegro OR cyprus OR libya OR lebanon OR egypt OR malta OR monaco OR slovenia OR syria OR tunisia OR turkey OR "Vatican City" OR greece OR spain OR israel OR italy OR portugal OR andorra OR "San Marino" OR kosovo OR serbia OR jordan OR sardinia OR “south europe” OR “south european” OR “southern european” OR “north Africa” OR “north African” OR “northern African” OR albanian OR bosnian OR gibraltarian OR algerian OR moroccan OR french OR croatian OR montenegrin OR cypriot OR libyan OR lebanese OR egyptian OR maltese OR monegasque OR slovenian OR syrian OR tunisian OR turkish OR vatican OR greek OR spanish OR israeli OR italian OR portuguese OR andorran OR sammarinese OR kosovar OR serbian OR jordanian OR sardinian OR medlife OR medis OR "Mediterranean Island Study" OR "Traditional Mediterranean lifestyle" OR tmedl OR "Seguimiento Universidad de Navarra" OR "SUN Cohort" OR "MOLI-SANI") AND (nap OR napp* OR siesta* OR messimeri OR "Quiet hour" OR "Quiet time" OR riposo OR qailulah OR "Midday Sleep" OR "Mid-day sleep" OR "Daytime Sleep" OR "Sleep Habits" OR "Mid-afternoon sleep" OR "Afternoon sleep" OR "Short sleep" OR "Daytime rest" OR "Brief sleep" OR "Catnap" OR "Mid-morning sleep" OR "Pre-evening sleep" OR "Restorative Sleep") AND (health* OR mortality OR death OR longevity OR "Life expectancy" OR survival OR "Life span" OR "Life lost" OR "Life loss" OR qualy* OR daly* OR qol* OR qual* OR "Diseas*" OR lifespan OR disorder* OR syndrome* OR deficit* OR dysfunction* OR morbidity OR hypertens* OR stroke* OR "Heart Failure" OR arrhythmia* OR diabet* OR immun* OR metaboli* OR obesity OR cancer* OR dementia* OR Alzheimer* OR sarcopenia OR osteoporosis OR gut OR inflammation OR cognitive OR neuro* OR "Self-harm" OR memor* OR frailty OR "Daily living" OR vitality OR anxiety OR depression OR resilien* OR autonom* OR burden OR life* OR aging OR ageing OR wellbeing OR wellness)
